# Fully Textile Dual-Band Logo Antenna for IoT Wearable Devices

**DOI:** 10.3390/s22124516

**Published:** 2022-06-15

**Authors:** Gabriela Lachezarova Atanasova, Blagovest Nikolaev Atanasov, Nikolay Todorov Atanasov

**Affiliations:** 1 Department of Communication and Computer Engineering, South-West University “Neofit Rilski”, 2700 Blagoevgrad, Bulgaria; natanasov@swu.bg; 2 91 German Language High School “Prof. Konstantin Galabov”, 1000 Sofia, Bulgaria; b_atanasov@outlook.com

**Keywords:** logo antenna, wearable antenna, fully textile antenna, IoT wearable device, RSSI, SAR

## Abstract

In recent years, the interest in the Internet of Things (IoT) has been growing because this technology bridges the gap between the physical and virtual world, by connecting different objects and people through communication networks, in order to improve the quality of life. New IoT wearable devices require new types of antennas with unique shapes, made on unconventional substrates, which can be unobtrusively integrated into clothes and accessories. In this paper, we propose a fully textile dual-band logo antenna integrated with a reflector for application in IoT wearable devices. The proposed antenna’s radiating elements have been shaped to mimic the logo of South-West University “Neofit Rilski” for an unobtrusive integration in accessories. A reflector has been mounted on the opposite side of the textile substrate to reduce the radiation from the wearable antenna and improve its robustness against the loading effect from nearby objects. Two antenna prototypes were fabricated and tested in free space as well as on three different objects (human body, notebook, and laptop). Moreover, in the two frequency ranges of interest a radiation efficiency of 25–38% and 62–90% was achieved. Moreover, due to the reflector, the maximum local specific-absorption rate, which averaged over 10 g mass in the human-body phantom, was found to be equal to 0.5182 W/kg at 2.4 GHz and 0.16379 W/kg at 5.47 GHz. Additionally, the results from the performed measurement-campaign collecting received the signal-strength indicator and packet loss for an off-body scenario in real-world use, demonstrating that the backpack-integrated antenna prototype can form high-quality off-body communication channels.

## 1. Introduction

Nowadays, the Internet of Things (IoT) has been identified as one of the leading technologies that bridges the gap between the physical and virtual world, by connecting different objects (from sensors and home electronics to clothing and accessories) and people through communication networks, in order to improve the quality of life [1,2,3,4,5,6,7,8,9]. For this reason, the IoT is used in many different areas such as finance, healthcare, sports, road-traffic management, education, and even agriculture. As a result, IoT devices are heterogeneous in appearance and functionality [10]. However, each wearable device generally includes one or more sensors, a processor, an operating system, a transceiver, and an antenna [11,12]. Such devices can be embedded in clothing or accessories, transmitting and receiving data wirelessly [11,13,14]. To be easily integrated into clothing and accessories, such as bags, backpacks, or pockets, wearable antennas must be flexible and light, which is why textile antennas are widely used in wearable devices [15,16,17,18]. Moreover, the antennas must be robust to human-body effects. As is well known, the proximity of the human body to an antenna can cause degradation of the radiation patter, input impedance matching, radiation efficiency, etc. Moreover, the radiation from wearable antennas should cause the least possible specific-absorption rate (SAR) inside the human tissue, in order to adhere to health and safety requirements [19,20]. Hence, it can be concluded that because wearable devices operate in specific environments (in most scenarios, close to the human body or other objects), the antennas of these devices are subject to specific requirements (such as mechanical, electrical, and safety, as detailed in [21]). 

According to an inquiry into the consumer IoT [13], ‘smart’ wearable devices are defined as one of the most widespread IoT devices among the citizens of the European Union, and forecasts show that the number of these devices will continue to increase. The need for more IoT devices has contributed to the development of new antenna topologies with unique shapes made on unconventional substrates. Unconventional substrates, such as polydimethylsiloxane polymer [22,23], natural rubber [24], and textiles [12,20,25,26,27,28,29,30,31,32], were used to create flexible antennas. Furthermore, for an unobtrusive integration into garments and accessories, several antenna geometries, such as those with buttons [33] or that are optically transparent [34], have a zip-based monopole [35], or are logo-based [36,37,38,39,40,41,42,43,44,45,46,47,48,49], have been reported. It should be noted that designing wearable textile antennas with a logo shape has several advantages. The first one is that the antennas can be placed anywhere on the garment or accessory, for example, on a pocket, jacket, shirt, etc. Another advantage of logo-shaped antennas is that they also can be used as advertising for a company or institution.

Most of the proposed textile logo-shaped antennas [36,38] have achieved good performance in the desired frequency band, but at the same time, due to their nearly omnidirectional radiation pattern, these antennas may increase SAR when working near the human body. Moreover, a detuning of the antenna parameters (such as resonant frequency, impedance matching, etc.) may take place due to the proximity of the human body or other objects. 

In this paper, we developed and realized a fully textile dual-band logo antenna integrated with a reflector for unobtrusive integration in clothes and accessories. Compared with other logo antennas on textile substrates, the proposed antenna shows the advantages of higher radiation efficiency and low SAR. Section 2 introduces the design and fabrication process of the proposed antenna. Section 3 presents the results from the numerical and experimental investigations of the parameters as well as the characteristics of the new logo-based antenna in both scenarios: in free space and when the antenna is placed on different objects. The conclusions are presented in Section 4.

## 2. Antenna Design and Fabrication Process 

### 2.1. Antenna Geometry

**Figure 1:**
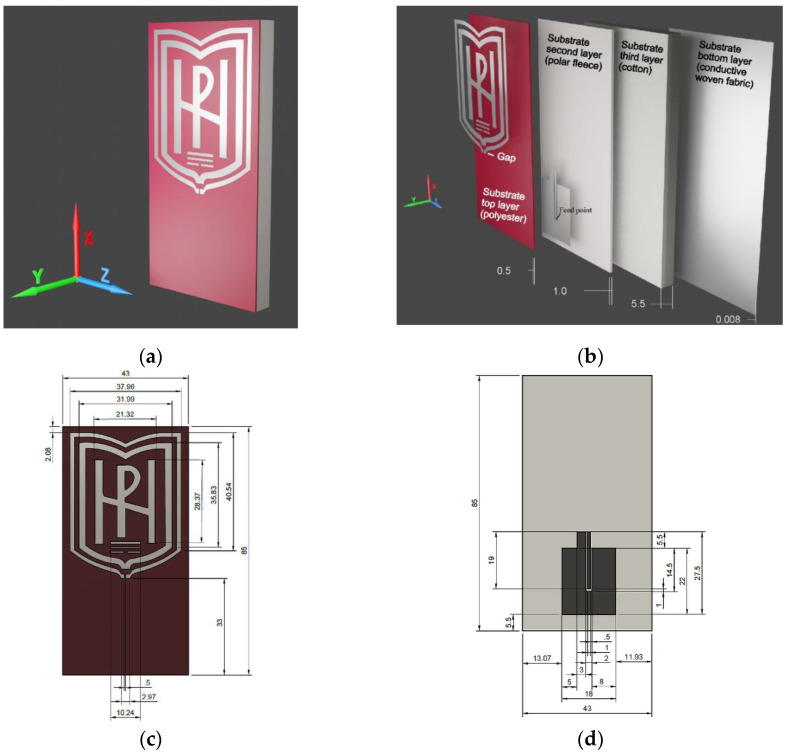
Configuration of the proposed antenna: (**a**) 3D view; (**b**) decomposition view; (**c**) top view with dimensions; (**d**) top view of substrate second layer with dimensions. Dimensions indicated in the figure are in millimeters.

**Figure 2:**
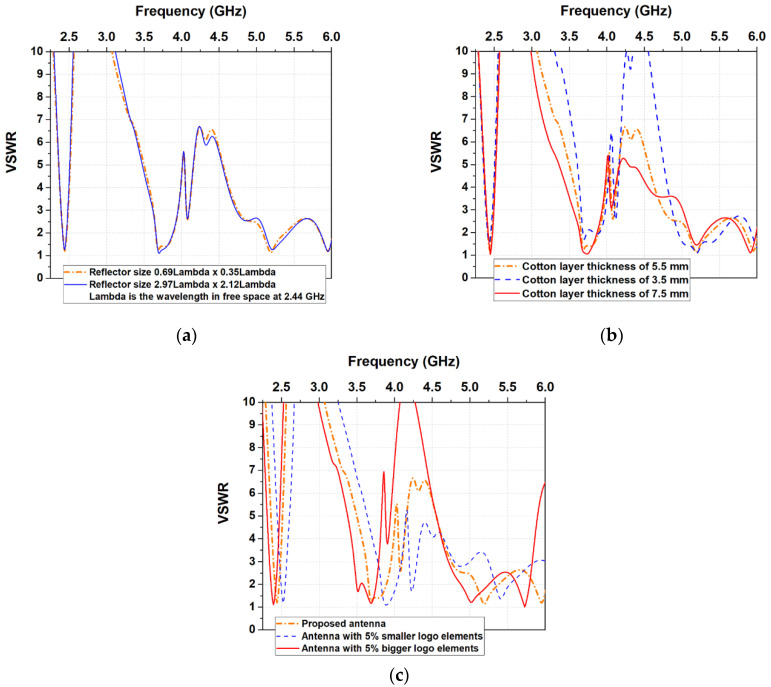
Voltage Standing Wave Ratio (VSWR) at a different: (**a**) reflector size; (**b**) cotton-layer thickness; (**c**) logo elements size. The dimensions of the other antenna elements are those shown in Figure 1. The reflector size is in wavelengths, where Lambda is the free-space wavelength at 2.44 GHz (central frequency for the ISM 2.4 GHz).

The proposed antenna consists of radiating elements, a three-layer textile substrate, and a reflector. The radiating structure of the antenna is based on the logo of South-West University “Neofit Rilski”, Blagoevgrad, Bulgaria. As can be seen from Figure 1a, the logo has a complex shape containing frames, letters, and disconnected parts. Due to this, the logo antenna is designed to operate as a loop antenna. The outer frame of the logo is used as the main radiating element, fed by a coplanar-waveguide (CPW) circuit. Initially, its dimensions (i.e., circumference of the loop) are set to approach one free-space wavelength (for the central frequency in the first frequency band), in order to obtain maximum radiation in the plane perpendicular to the loop [50]. To get a clean logo on a garment or accessory, we mounted the CPW on the second layer of the substrate and fed the antenna through a gap in the substrate top layer, as shown in Figure 1b. In the next step in the antenna-design process, the radiating logo elements are placed above a reflector to maintain undisturbed antenna impedance, when the wearable device is mounted on a human body or another medium. The reflector is separated from the CPW by a textile substrate (polar fleece and cotton). We have found through numerous simulations that to achieve maximum bandwidths, radiation efficiency, and good impedance matching with minimum antenna size and profile, the reflector size should be approximately 0.69λ × 0.35λ, and the cotton-layer thickness should be approximately 0.057λ (λ is the free-space wavelength at 2.44 GHz). As shown in Figure 2a and Table 1, using a larger reflector would provide a better front-to-back ratio (FBR), without significantly affecting the VSWR (respectively, input impedance behavior, and resonant frequencies) and radiation efficiency. On the other hand, as shown in Figure 2b and Table 2, when cotton-layer thickness decreases, the radiation efficiency and FBR also decrease. By choosing a substrate thickness of 7 mm, a compromise is reached between the antenna profile and the high radiation efficiency in the desired frequency bands. In addition, since the antenna is intended primarily for easy integration in accessories, the substrate thickness does not affect its installation in a backpack, handbag, or other similar accessory, as demonstrated in the article. The changes in the dimensions of the logo elements give a more pronounced effect on the VSWR in the first frequency band. As can be observed from Figure 2c, the resonant frequency in each of the three frequency bands shifts to the left (to lower frequencies), as the size of the logo elements increases. In contrast, the resonant frequency in each of the three frequency bands shifts to the right (to higher frequencies), as the size of logo elements decreases. The dimensions of the antenna elements are tuned to achieve optimal impedance matching and radiation efficiency in the frequency bands: 2.4–2.48 GHz (Industrial, Scientific, and Medical (ISM)), 5.150–5.850 GHz Unlicensed National Information Infrastructure (U-NII (U-NII-1 to U-NII-3)) and 5.75–5.82 GHz (ISM). These frequency bands have been chosen because most IoT wearable devices use wireless technologies such as Bluetooth, Wi-Fi, IEEE 802.11ah, IEEE 802.15.4, and Zigbee, in order to interact, collect, and exchange data [51,52]. From Figure 2a, it is seen that the antenna also covers the 3.4–3.6 GHz frequency band (Worldwide Interoperability for Microwave Access—WiMAX). It will not be examined here because it is not applicable to wearable devices. 

### 2.2. Materials and Methods

#### 2.2.1. Materials

This paper aims to realize an aesthetic logo-based antenna made entirely of textile materials, for an unobtrusive integration into a garment or an accessory. A highly flexible and conductive woven fabric P1168 (Adafruit, New York, NY, USA), with a thickness of 8 μm, was selected for the conductive parts of the antenna. For the antenna’s substrate, among the available non-conductive fabrics, we chose two knitted fabrics (polyester and cotton) and a non-woven fabric (polar fleece). A polymer strip activated by ironing is used to attach the antenna elements. Table 3 presents the thickness and electromagnetic properties (real part of the complex permittivity (ε^|^) and conductivity (σ)) of the fabrics used in the antenna substrate. 

For assessing the antenna performance near a notebook and laptop, electromagnetic properties of copy-printer paper (80 g/m^2^) and plastic were measured. The results were used to prepare numerical models.

#### 2.2.2. Methods and Models

The electromagnetic properties of the materials listed in Table 3 and Table 4 were measured at a frequency of 2.56 GHz by the resonant perturbation method, as described in [21,53].

The design, optimization, and numerical investigations of the antenna parameters and characteristics were performed using Remcom xFDTD finite-difference time-domain (FDTD) commercial software. In all of the simulations, two FDTD space lattices were applied: (1) a fine uniform cubic FDTD lattice with cell size from 0.5 mm for the antenna elements; and (2) a non-uniform FDTD lattice having an increasing cell size from 0.5 mm to 1 mm for the rest of the space. The FDTD space-lattice resolutions were defined by the textile thickness used for the substrate. A 7-layer perfectly matched layer-absorbing boundary condition was used on all boundaries. The total simulation required 30,000 time-steps.

For creating the numerical model of the antenna, the logo of South-West University “Neofit Rilski” was first designed using the cloud-based CAD software platform Fusion 360. After that, the CAD dataset was exported in IGS format to FDTD software and the logo elements were modeled as a perfect electric conductor (PEC). Once the numerical model of the South-West University “Neofit Rilski”-logo was created in FDTD, a three-layer substrate was added and positioned under the logo elements with the electromagnetic parameters listed in Table 3. A numerical model of CPW was created on the second substrate layer, and a feeding port was inserted. On the backside of the substrate, a reflector was added and modeled as PEC. 

Three numerical models were developed to mimic a human body, notebook, and laptop. First, a homogenous numerical model (flat phantom) with the dimensions and electromagnetic properties presented in Table 4 was developed to mimic the human body. A homogenous model, which represents the notebook, also was created. Finally, a three-layer model consisting of two layers of plastic and one layer of metal was designed to represent the laptop. For each model, the electromagnetic properties, dimensions, and mass density values are listed in Table 4. 

### 2.3. Antenna Prototype Fabrication Process

**Figure 3:**
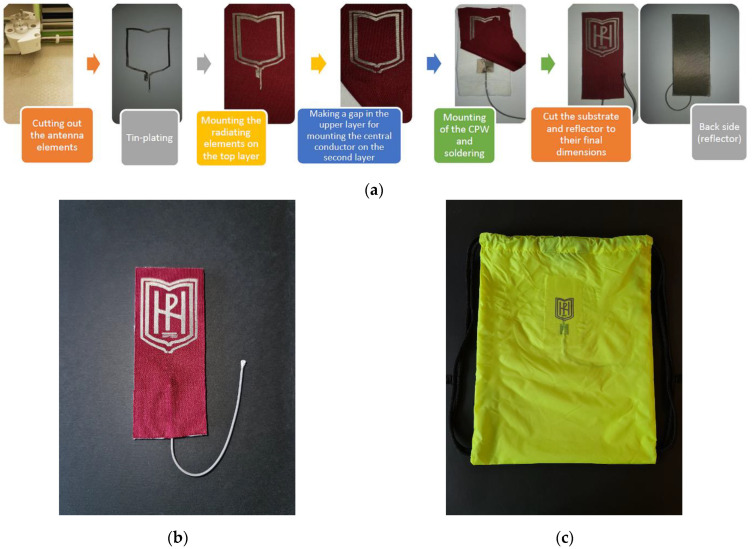
Antenna prototype fabrication process: (**a**) block diagram; (**b**) photograph of the fabricated prototype 1, and (**c**) photograph of the fabricated prototype 2—integrated into a backpack.

The fabrication of the proposed logo antenna was accomplished layer by layer. The block diagram of the fabrication process is shown in Figure 3a. First, the logo elements and CPW were directly shaped on the conductive fabric with a high accuracy of 0.01 mm using a cutting machine (Cricut Explore Air 2, Provo Craft & Novelty Inc., South Jordan, UT, USA). Next, the soldering points of the woven fabric with the CPW and the coaxial cable were tin-plated at 180 °C. After that, the substrate (consisting of: one layer (0.5 mm) of polyester, one layer (1.0 mm) of polar fleece, and seven layers (0.786 mm each) of cotton fabric) and a reflector were cut to have a rectangular form. The antenna-radiating elements were mounted on the top layer. Afterwards, the cotton and polar-fleece layers were attached to the reflector. As can be seen in the block diagram, a slot was made in the top layer, and the central conductor of the CPW line was mounted on the second layer. After that, a 1.13 mm mini-coaxial cable with a U.FL connector was soldered to the CPW and fixed with thread to the polar fleece, as shown in Figure 3a. All antenna elements were assembled by hand using polymer tape (SY, IKEA) that was activated by ironing. Finally, the antenna substrate and reflector were cut to the dimensions shown in Figure 1. Figure 3b shows the fabricated prototype of the antenna. Moreover, to verify the antenna performances in real-world use, the proposed antenna was fabricated and unobtrusively integrated into a backpack, as shown in Figure 3c.

## 3. Results and Discussions

In this section, we present the results from the numerical and experimental investigations of the performance of the logo-based antenna in two environments: (1) free space and (2) in the presence of the models of a human body, notebook, and laptop, in order to determine the effects of these objects on antenna performance. 

### 3.1. Simulated Results

#### 3.1.1. Reflection Coefficient

**Figure 4:**
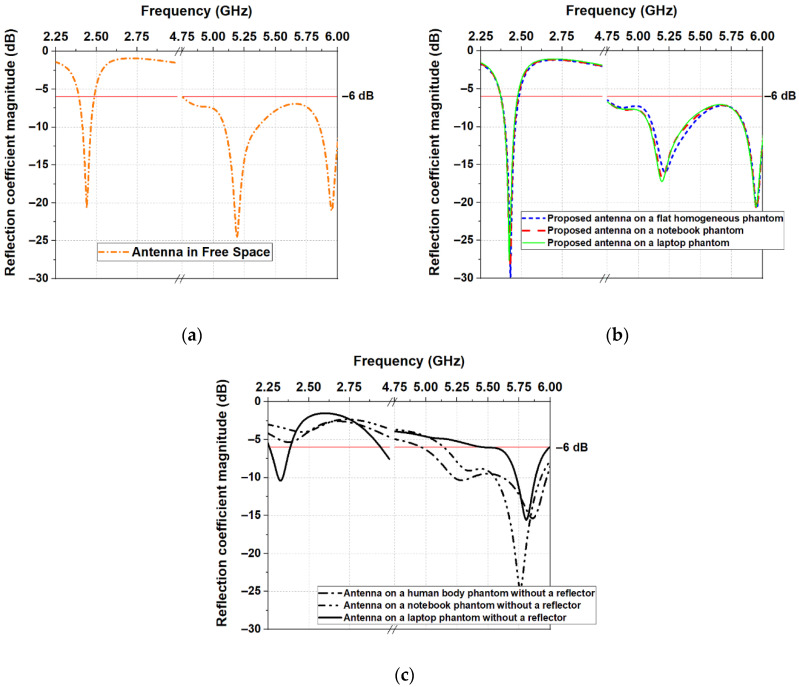
Simulated reflection coefficient |S_11_| of the proposed antenna: (**a**) in free space; (**b**) on a model of a human body, notebook, and laptop; (**c**) antenna without a reflector on a model of a human body, notebook, and laptop.

The predicted reflection coefficient |S_11_| of the antenna in free space, as a function of frequency, is shown in Figure 4. As can be seen, the antenna is tuned to operate at two frequency bands, where |S_11_| is less than −6 dB. A −6 dB |S_11_| reference level (VSWR ≤ 3) is used to define the impedance bandwidth, which is usually accepted for small antennas (such as handset and wearable antennas) [54,55,56] and multiband (multi-resonance) antenna [57]. The first frequency band is from 2.393 GHz to 2.488 GHz, and the second one is from 4.75 GHz to 6.0 GHz, both covering the entire ISM 2.4 GHz, from U-NII-1 to the U-NII-3 and ISM 5 GHz bands. Consequently, the antenna can be used for multi-band applications.

In order to investigate the effects of different objects on the performance of the proposed antenna, we performed full-wave simulations by placing the antenna on three different models: (1) a flat homogeneous phantom representing a human body, (2) a notebook model, and (3) a laptop model. Figure 4b shows the simulated |S_11_| of the antenna on the three numerical models. 

When comparing the results depicted in Figure 4a,b, we can see that the resonant frequencies remain unchanged. Moreover, placing the antenna on models of the human body, notebook, and laptop leads to minor changes in |S_11_|. These results confirm that the presence of the reflector isolates the antenna from the lossy medium, allowing for maintaining the undisturbed antenna impedance when the wearable device is placed on a human body or another medium, such as a human body, notebook, or laptop. Moreover, comparing the simulated S-parameters of the proposed antenna with those of the antenna without a reflector (Figure 4c), we can see a significant shift in resonant frequencies when the antenna without a reflector is placed on different objects. Consequently, due to the good insolation provided by the reflector, the proposed logo antenna can operate on different surfaces, retaining its resonant frequencies and impedance-matching properties. 

#### 3.1.2. Radiation Patterns

**Figure 5:**
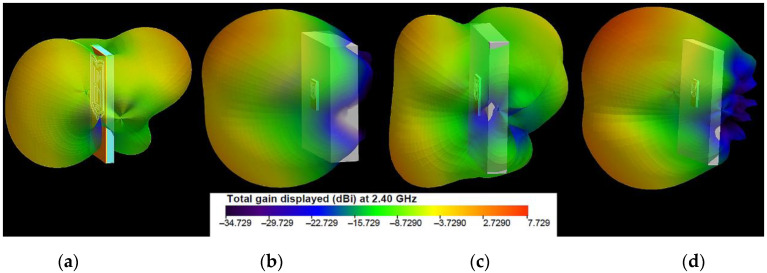
Simulated 3D radiation patterns of the antenna at 2.40 GHz: (**a**) in free space; (**b**) on the human-body model; (**c**) on the notebook model; (**d**) on the laptop model.

**Figure 6:**
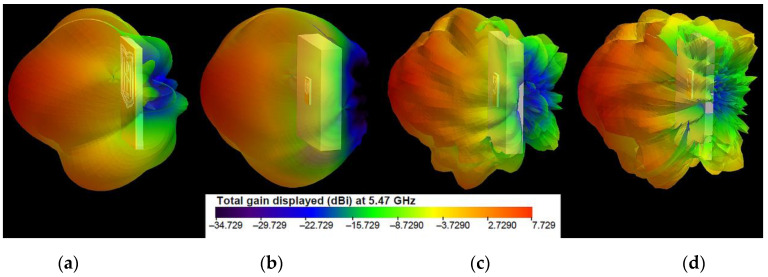
Simulated 3D radiation patterns of the antenna at 5.47 GHz: (**a**) in free space; (**b**) on the human-body model; (**c**) on the notebook model; (**d**) on the laptop model.

The three-dimensional (3D) radiation patterns of the proposed antenna in free space and on the models of the human body, notebook, and laptop at 2.4 GHz and 5.47 GHz are shown in Figure 5 and Figure 6, respectively. 

**Figure 7:**
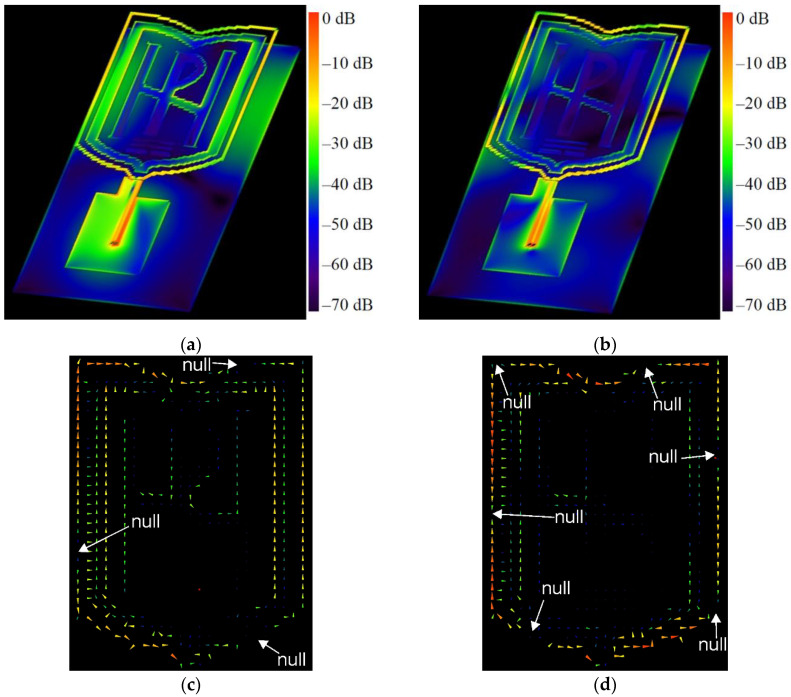
Normalized current distribution on the: (**a**) logo elements, CPW, and reflector at 2.4 GHz; (**b**) logo elements, CPW, and reflector at 5.47 GHz; (**c**) logo elements at 2.4 GHz; (**d**) logo elements at 5.47 GHz.

The results show that when the antenna is in free space, at 2.4 GHz, it produces dipole-like radiation. The radiation-pattern maximum is in the plane perpendicular to the antenna because the circumference of the outer logo frame is about 1.25λ at 2.4 GHz, while the perimeter of the inner frame approaches one free-space wavelength at 2.4 GHz. Figure 7 shows the currents excited on the antenna elements. As we can see, the surface currents for the two frequencies are fewer on the reflector and mainly distributed on the logo elements and around the CPW. For 2.4 GHz excitation, a large surface-current distribution is observed over the logo frames and on the edges of the reflector, enhancing radiation. The surface currents on only the logo elements are given in Figure 7c,d. The currents are presented in the form of vectors, in order to identify the nulls. From the results in Figure 7c, it can be seen that the surface currents on the outer logo frame at 2.4 GHz show three current nulls, which confirms again that the antenna works at a loop basic (with about 1.25λ loop) mode. The surface-current density and the directions of the current vectors on the inner logo frame are the same as those on the outer logo frame. At 5.47 GHz, the six nulls are formed on the outer logo frame, as shown in Figure 7d. Consequently, the antenna works at about the 3-wavelength mode. Moreover, from Figure 7b, it can be seen that at 5.47 GHz there are small excited surface-current distributions on the reflector. From Figure 6b, we can also see that the directivity increases as the frequency increases. Moreover, as the frequency increases, the current amplitude along the frames becomes lower. Furthermore, the directivity increases when the wearable antenna is placed on the phantoms. The results in Figure 6 indicate that, at 5.47 GHz, the wearable antenna produces patch-like radiation, which is desirable for off-body communications [16].

#### 3.1.3. Gain, Efficiency, and Front-to-Back Ratio

**Figure 8:**
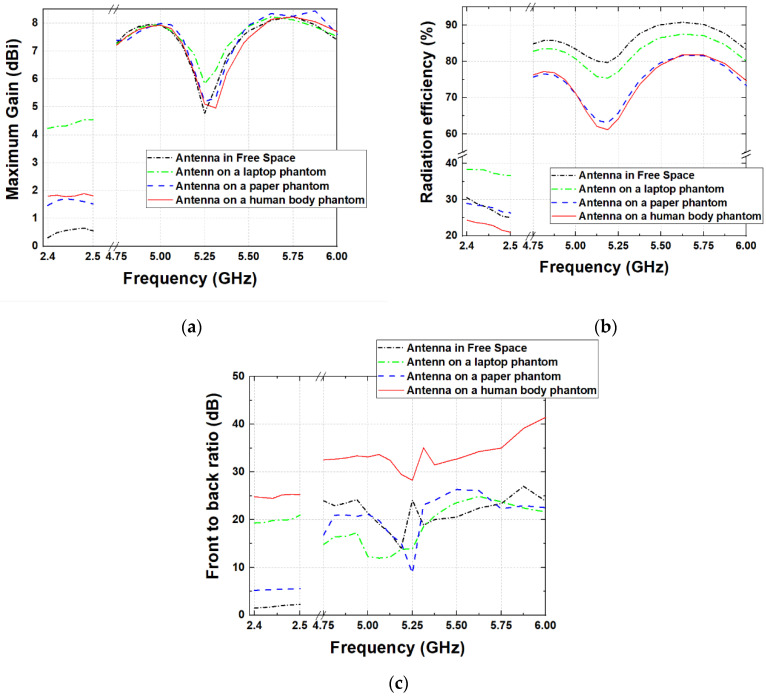
Simulated: (**a**) maximum gain; (**b**) radiation efficiency versus frequency; (**c**) FBR.

The variation of gain, radiation efficiency, and FBR of the proposed fully textile dual-band logo antenna in free space and on the models of the human body, notebook, and laptop are shown in Figure 8. 

The results from numerical simulations show that, in the frequency range from 2.4 GHz to 2.5 GHz, the gain characteristics of the proposed antenna are slightly influenced by the presence of the numerical models of the human body, notebook, and laptop. As seen in Figure 8a, with the presence of a phantom, the maximum gain is increased from 0.5 dBi (in free space) to 1.6–1.7 dBi (on the paper and human-body phantoms) and 4.5 dBi (on the laptop phantom). The variation in the gain in the first frequency range is a consequence of the reflector size (0.69λ × 0.35λ at the central frequency). When the antenna is mounted on a phantom, the size of the reflector is small compared to the phantom size. As a result, a part of the radiated energy is directed toward the phantom and undergoes a reflection. The increase in the gain is due to the in-phase reflection from the phantoms. From the results, we also can see that there are no variations in the gain characteristics of the proposed antenna in the frequency range from 4.75 GHz to 6.0 GHz, when the antenna is in free space and when it is placed on the models of a human body, notebook, and laptop. This is due to the fact that in the second frequency band, the size of the reflector is 1.52λ × 0.77λ at the central frequency.

The results in Figure 8b show that, in the frequency range of 2.4 GHz to 2.5 GHz, the antenna-radiation efficiency decreases slightly with increasing frequency. Moreover, the radiation efficiency is slightly reduced (between 1–5%) when the antenna is placed on the notebook and human-body phantoms. The slight reduction in the radiation efficiency is due to the fact that a portion of the antenna-delivered power is absorbed in the phantom when the antenna is located on a lossy medium [54]. The highest efficiency is achieved when the antenna is placed on the laptop-numerical model. Furthermore, in the frequency range of 4.75 GHz to 6.0 GHz, efficiencies of the order of 62–90% are achieved. 

Figure 8c shows the FBR as a function of the frequency. As can be seen, due to the size of the reflector in the 2.4–2.5 GHz frequency band, the FBR in free space is about 2 dB, while across 4.75–6 GHz, it is greater than 15 dB. The results also show that when the antenna is placed on the human-body phantom, the FBR is greater than 25 dB across 2.4–2.5 GHz and is greater than 30 dB across 4.75–6 GHz. This is due to the dimensions and electromagnetic properties of the phantom. Moreover, when the antenna is placed on the paper or laptop phantom, the FBR is greater than 10 dB across 4.75–6 GHz.

#### 3.1.4. Specific-Absorption Rate

**Figure 9:**
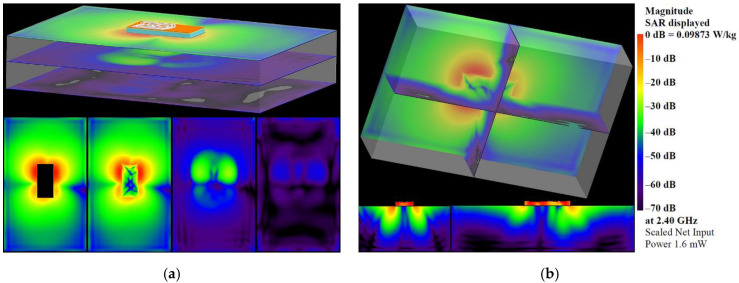
SAR distributions in: (**a**) different planes; (**b**) different cross-sections at 2.4 GHz.

**Figure 10:**
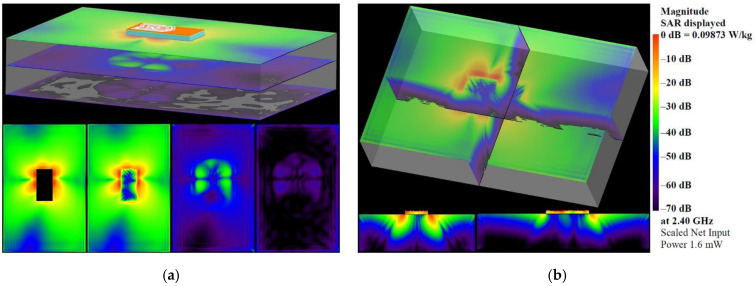
SAR distributions in: (**a**) different planes; (**b**) different cross-sections at 5.47 GHz.

Simulated SAR distributions in different planes and cross-sections at 2.4 GHz and 5.47 GHz are presented in Figure 9 and Figure 10 to evaluate human exposure due to the electromagnetic fields generated from the proposed antenna. The SAR was calculated at the position representing the conditions of the normal use of the antenna, i.e., when it is embedded into garments or accessories worn by a person. Moreover, to highlight the visibility of the lowest SAR values, a logarithmic scale (with a range of 70 dB) is used. As can be seen from the SAR distributions, the highest SAR values are concentrated around the antenna-radiating elements. Below the antenna, the SAR values are 30 dB lower. Moreover, SAR values fall off rapidly with increasing distance from the antenna. As can be seen from Figure 9 and Figure 10, the SAR values in the plane located 35 mm below the antenna are 45–50 dB lower. The maximum local SAR averaged over 10 g mass at a net input power of 100 mW, is found to be equal to 0.5182 W/kg at 2.4 GHz and 0.16379 W/kg at 5.47 GHz. These values are 75% and 92% lower than the 10 g SAR limit of 2 W/kg, set by the international standards and guidelines [58,59].

### 3.2. Measured Results

**Figure 11:**
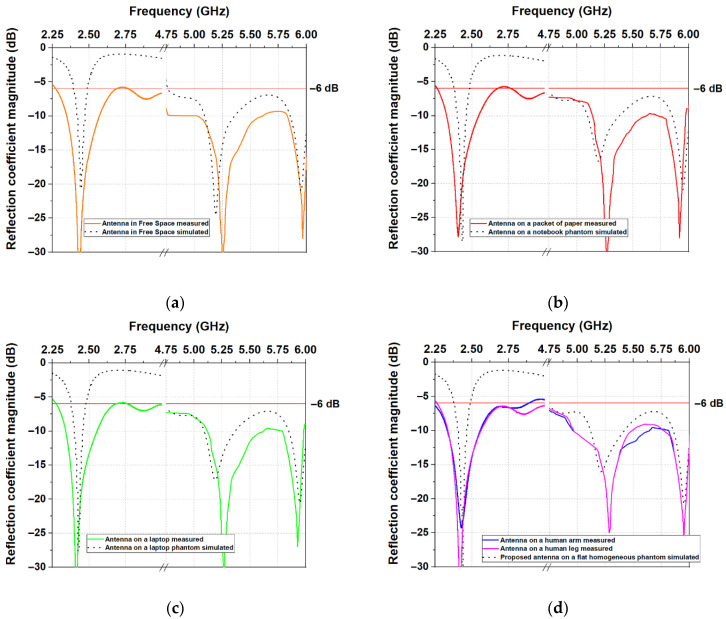
Measured and simulated reflection coefficient |S_11_|: (**a**) in free space; (**b**) on the copy-printer paper; (**c**) on the laptop; (**d**) on the human arm, leg, and flat phantom.

The measured and simulated reflection coefficient |S_11_| of the antenna, as a function of frequency, is shown in Figure 11. The experimental results reveal that placing the antenna on different objects (such as a human arm and leg, laptop, or paper) does not change the resonant frequency. Moreover, a good agreement can be found between the experimental and predicted results. Comparing the measured |S_11_| to the predicted results by simulation, we can see that there are no variations in the resonance frequency. Furthermore, a bandwidth broadening is observed in the first bandwidth of interest.

### 3.3. Comparison

A performance comparison between the proposed fully textile wearable antenna and those textile wearable antennas previously published in the literature, concerning their radiation efficiency and SAR, is depicted in Table 5. It can be seen that the proposed antenna has two main advantages compared to the antennas in [19,20,22,23,27,28,29,30,31,32,35,36,41,43,51]. The primary advantage of the proposed antenna structure is that it provides the highest radiation efficiency both in free space and on a human-body phantom in the ISM 5.8 GHz band (5.725 GHz–5.875 GHz). Another advantage is that the proposed fully textile wearable antenna exhibits lower SAR than wearable antennas with textile substrates [20,27,28,29,30,31,32]. Therefore, the proposed antenna structure is suitable for IoT wearable devices that can be placed on the human body.

### 3.4. Applications

**Figure 12:**
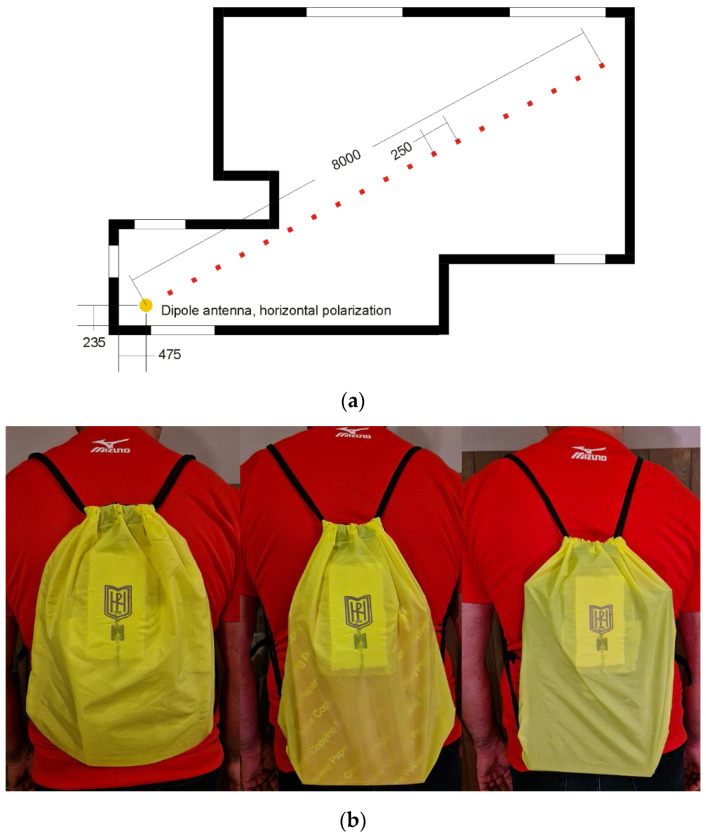
Measurement setup: (**a**) floor plan of the measurement locations and (**b**) photographs of the empty backpack, backpack with a package of copy-printer paper, and backpack with a laptop. The dimensions indicated in the figure are in millimeters.

**Figure 13:**
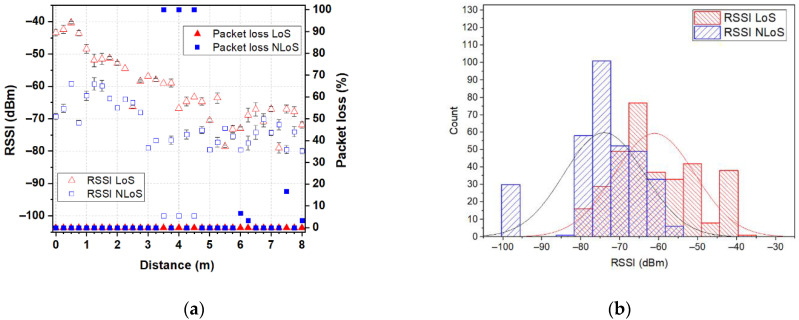
RSSI and packet loss for the backpack-integrated antenna prototype in the case of an empty backpack: (**a**) distributions with respect to the distance between the antennas; (**b**) histograms. The RSSI and packet loss for all positions in the LoS scenario are displayed with red triangles. The RSSI and packet loss for all positions in the NLoS scenario are displayed with blue squares.

**Figure 14:**
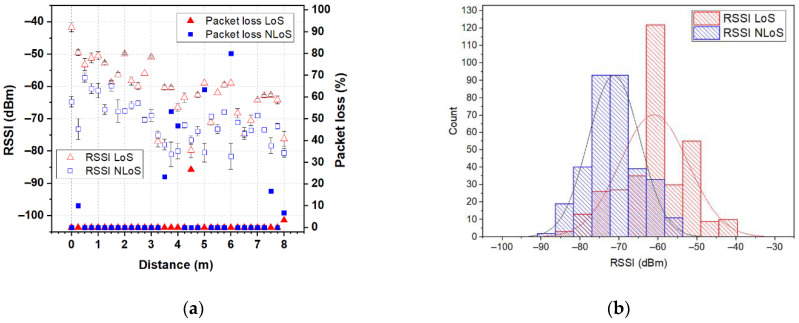
RSSI and packet loss for the backpack-integrated antenna prototype in the case of a backpack with a package of copy-printer paper: (**a**) distributions with respect to the distance between the antennas; (**b**) histograms. The RSSI and packet loss for all positions in the LoS scenario are displayed with red triangles. The RSSI and packet loss for all positions in the NLoS scenario are displayed with blue squares.

**Figure 15:**
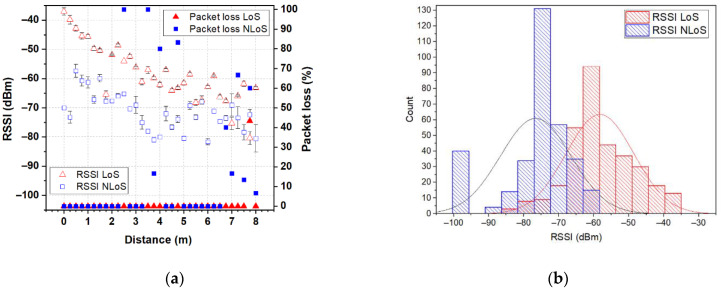
RSSI and packet loss for the backpack-integrated antenna prototype in the case of a backpack with a laptop: (**a**) distributions with respect to the distance between the antennas; (**b**) histograms. The RSSI and packet loss for all positions in the LoS scenario are displayed with red triangles. The RSSI and packet loss for all positions in the NLoS scenario are displayed with blue squares.

In order to demonstrate the feasibility of the proposed antenna, for unobtrusive integration into accessories for use in IoT wearable applications that provide environmental condition monitoring or a safe environment for the users, we performed a measurement campaign collecting received signal-strength indicator (RSSI) and packet loss from real-world use. Two XBee 802.15.4 modules (transmit power 1 mW, receiver sensitivity −100 dBm) marked as XBee A and XBee B were used to represent the on- and off-body nodes in a wireless body-area network. In the measurement campaign, the backpack-integrated antenna prototype was connected to the XBee A module and put on a test person’s back in order to represent the on-body node. A dipole antenna (resonant frequency 2.44 GHz, bandwidth 615 MHz at |S_11_| ≤ −6 dB) in a horizontal orientation connected to the XBee B module was used to represent the off-body node (coordinator). The XBee B module was connected to a laptop through a UART-to-USB controller and was programmed to operate at 2.41 GHz (C channel) using X-CTU software. The measurements were conducted in a residential environment for line-of-site (LoS) and non-line-of-site (NLoS) scenarios, as shown in Figure 12a. The test person with the backpack-integrated antenna prototype stood alternatively in LoS (with their backside to the dipole antenna) and NLoS (facing the dipole antenna) conditions. The measurements start from 0 mm from the dipole antenna and have been repeated every 250 mm (two wavelengths at 2.45 GHz), up to a distance of 8 m from the coordinator. For each of the 33 antenna locations, 10 measurements of RSSI and packet loss were conducted. Figure 13, Figure 14 and Figure 15 show the RSSI and packet-loss distributions, with respect to the distance between the antennas in three cases: empty backpack, backpack with a package of copy-printer paper, and backpack with a laptop (Figure 12b). 

From the results, it can be observed that in the LoS scenario when antennas are at a distance of 0.0 m (the backpack-integrated antenna prototype is 0.4 m above the dipole antenna) from each other the RSSI is about −43 dBm for the case of the empty backpack, −42 dBm and −37 dBm for cases of the backpack in which are placed a package of copy-printer paper and laptop, respectively. Furthermore, when antennas are at 8 m from each other, the RSSI values are between −60 dBm and −75 dBm. The lowest RSSI values are between −80 and −85 dBm, respectively. Moreover, the results show that the proposed logo antenna is able to perform off-body communications without packet loss at an 8 m distance from the coordinator node.

Another observation that could be done is that in the NLoS scenario, the RSSI values are smaller than in the LoS scenario for each of the three cases (empty backpack, backpack with a package of copy-printer paper and backpack with a laptop), due to the shadowing effect of the human body. In the NLoS scenario, there are several positions where packet loss is observed. This can be explained by the fact that in this scenario the propagation occurs basically by reflection on the surrounding objects (such as walls, tables, etc.). Moreover, when antennas are 8 m from each other, the RSSI is about −80 dBm for each of the three cases. These results reveal that the proposed fully textile logo antenna is suitable for off-body IoT wearable wireless applications.

## 4. Conclusions

This paper demonstrates a new fully textile dual-band logo antenna integrated with a reflector for application in IoT wearable devices. The proposed antenna’s radiating elements have been shaped to mimic the logo of South-West University ‘‘Neofit Rilski’’. This approach allowed us to unobtrusively integrate the antenna into a backpack and test its performances in real-world use in a wireless Zigbee network. Moreover, the mounted reflector on the opposite side of the textile substrate allows for reducing the radiation from the wearable antenna and improves its robustness against the loading effect from nearby objects. The numerical and experimental investigations of the antenna performance in two environments: (1) in free space and (2) on models of a human body, notebook, and laptop, reveal that the proposed fully textile logo antenna achieved radiation efficiency between 25–30% (in free space) and 20–38% (on the human body, paper, and laptop phantoms) in the frequency range from 2.4 GHz to 2.5 GHz. In the frequency range from 4.75 GHz to 6 GHz, an efficiency of 62–90% was achieved. Moreover, due to the reflector, the maximum local SAR averaged over 10 g mass in the human-body phantom was found to be equal to 0.5182 W/kg at 2.4 GHz and 0.16379 W/kg at 5.47 GHz. Additionally, the results from the performed measurements of RSSI and packet loss in an indoor environment, when the backpack integrated logo antenna is placed on a human back, demonstrate that the antenna can form high-quality off-body communication channels for the LoS and NLoS scenarios.

## Figures and Tables

**Table 1:** Antenna parameters at different reflector sizes (the cotton layer and logo elements are with the dimensions shown in Figure 1c).

Reflector Size ^1^	Frequency ^2^	VSWR	FBR ^3^	Rad. Eff. ^4^
0.69λ × 0.35λ	2.40	2.63	1.51	30.55
2.97λ × 2.12λ	5.47	2.25	20.40	89.80
0.69λ × 0.35λ	2.40	2.58	28.03	29.66
2.97λ × 2.12λ	5.47	2.12	30.93	90.29

^1^ The reflector size indicated in the table is in wavelengths, where λ is the free-space wavelength at 2.44 GHz (central frequency for the ISM 2.4 GHz). ^2^ The frequency indicated in the table is in GHz. ^3^ The front-to-back ratio (FBR) indicated in the table is in dB. ^4^ The radiation efficiency (Rad. Eff.) indicated in the table is in %.

**Table 2:** Antenna parameters at different cotton-layer thicknesses. The dimensions of the other antenna elements are those shown in Figure 1.

Cotton-Layer Thickness ^1^	Frequency ^2^	VSWR	FBR ^3^	Rad. Eff. ^4^
3.5	2.40	2.48	1.47	23.73
3.5	5.47	1.71	22.94	88.07
5.5	2.40	2.63	1.51	30.55
5.5	5.47	2.25	20.40	89.80
7.5	2.40	2.97	2.11	37.70
7.5	5.47	2.52	19.04	90.52

^1^ The cotton-layer thickness indicated in the table is in millimeters. ^2^ The frequency indicated in the table is in GHz. ^3^ The front to back ratio (FBR) indicated in the table is in dB. ^4^ The radiation efficiency (Rad. Eff.) indicated in the table is in %.

**Table 3:** Material properties of the fabrics used in the antenna substrate.

Materials	Thickness ^1^	ε^|^	σ ^2^	Density ^3^
Polyester	0.5	1.49797	0.000852	1380
Polar fleece	1	1.06615	0.000676	200
Cotton	5.5	1.58710	0.010976	1520

^1^ The thickness indicated in the table is in millimeters and corresponds to one layer of the substrate. ^2^ The conductivity indicated in the table is in S/m. ^3^ The density indicated in the table is in kg/m^3^.

**Table 4:** Material properties of the models used for investigation of the effects of different objects on the performance of the proposed antenna.

Numerical Models	Dimensions ^1^	ε^|^	σ ^2^	Density ^3^
Human body (flat phantom)	340 × 220 × 70	40.805	2.33000	1166
Copy-printer paper	297 × 210 × 50	2.555	0.04588	800
Laptop plastic box	365 × 260 × 30	2.789	0.01600	800

^1^ The dimensions indicated in the table are in millimeters. ^2^ The conductivity indicated in the table is in S/m. ^3^ The density indicated in the table is in kg/m^3^.

**Table 5:** Comparison of the proposed antenna.

References	Proposed Antenna	[19]	[20]	[22]	[23]	[27]	[28]	[29]	[30]	[31]	[32]	[35]	[36]	[41]	[43]	[51]
RE_FB_ FS ^1^	30.55	-	60	63.5	-	-	-	-		-	-	-		15.2	-	66
RE_SB_ FS ^2^	90.82	-	-	53	42	-	-	-	74.1	85	-	64	66	41.4	62	78
RE_FB_ HBP ^3^	24.36	-	20	58.6	-	-	-	12		-	-	-	-	-		-
RE_SB_ HBP ^4^	81.88	-	-	50.6	40	-	-		64.2	19	-	-	-	-	-	-
SAR_FB_ 10 g ^5^	0.518	0.26	1.5	-	-	3.2	1.14	1.96		-	1.33	-	-	-	-	-
SAR_SB_ 10 g ^6^	0.164	-	-	-	-	-	-		0.41	0.5	-	-	-	-	-	-

^1^ Radiation efficiency (RE) in free space (FS) at ISM 2.4 GHz. ^2^ Radiation efficiency in free space at ISM 5.8 GHz. ^3^ Radiation efficiency when the antenna is on a human-body phantom (HBP) at ISM 2.4 GHz. ^4^ Radiation efficiency when the antenna is on a human-body phantom at ISM 5.8 GHz. ^5^ Maximum local SAR averaged over 10 g mass at a net input power of 100 mW, when the antenna is on a human-body phantom at ISM 2.4 GHz. ^6^ Maximum local SAR averaged over 10 g mass at a net input power of 100 mW, when the antenna is on a human-body phantom at ISM 5.8 GHz.

## References

[1] Al-Sehemi A.G., Al-Ghamdi A.A., Dishovsky N.T., Atanasov N.T., Atanasova G.L. (2021). Design of a flexible waterproof antenna for Internet of Things applications. J. Electromagn. Waves Appl..

[2] Al-Sehemi A., Al-Ghamdi A., Dishovsky N., Atanasova G., Atanasov N. (2020). A flexible broadband antenna for IoT applications. Int. J. Microw. Wirel. Technol..

[3] Gómez M.E.D.C., Álvarez H.F., Berdasco A.F., Andrés F.L.-H. (2020). Paving the Way to Eco-Friendly IoT Antennas: Tencel-Based Ultra-Thin Compact Monopole and Its Applications to ZigBee. Sensors.

[4] Jagan G.C., Jayarin P.J. Modern Resource Conservation Strategies to Develop Multifaceted Applications of Wireless Sensor Networks: A Review. Proceedings of the 2022 International Conference on Communication, Computing and Internet of Things (IC3IoT).

[5] Al-Fuqaha A., Guizani M., Mohammadi M., Aledhari M., Ayyash M. (2015). Internet of Things: A Survey on Enabling Technologies, Protocols, and Applications. IEEE Commun. Surv. Tutor..

[6] Abbas N., Zhang Y., Taherkordi A., Skeie T. (2018). Mobile Edge Computing: A Survey. IEEE Internet Things J..

[7] Sisinni E., Saifullah A., Han S., Jennehag U., Gidlund M. (2018). Industrial Internet of Things: Challenges, Opportunities, and Directions. IEEE Trans. Ind. Inform..

[8] Ali M.U., Mishra B.K., Thakker D., Mazumdar S., Simpson S. (2021). Using Citizen Science to Complement IoT Data Collection: A Survey of Motivational and Engagement Factors in Technology-Centric Citizen Science Projects. IoT.

[9] Poulter A.J., Cox S.J. (2021). Enabling Secure Guest Access for Command-and-Control of Internet of Things Devices. IoT.

[10] Desai B.A., Divakaran D.M., Nevat I., Peter G.W., Gurusamy M. A feature-ranking framework for IoT device classification. Proceedings of the 2019 11th International Conference on Communication Systems & Networks (COMSNETS).

[11] Al-Sehemi A., Al-Ghamdi A., Dishovsky N., Atanasova G., Atanasov N. (2017). A Flexible Planar Antenna on Multilayer Rubber Composite for Wearable Devices. Prog. Electromagn. Res. C.

[12] Loss C., Gonçalves R., Lopes C., Pinho P., Salvado R. (2016). Smart Coat with a Fully-Embedded Textile Antenna for IoT Applications. Sensors.

[13] Commission Staff Working Document. Preliminary Report—Sector inquiry into Consumer Internet of Things. Proceedings of the European Commission.

[14] Alhmiedat T., Aborokbah M. (2021). Social Distance Monitoring Approach Using Wearable Smart Tags. Electronics.

[15] Sreelakshmy R., Kumar S.A., Shanmuganantham T. (2017). A wearable type embroidered logo antenna at ISM band for military applications. Microw. Opt. Technol. Lett..

[16] Kumar V. Logo based dipole antenna for RFID applications. Proceedings of the 2017 International Conference on Energy, Communication, Data Analytics and Soft Computing (ICECDS).

[17] Dang Q.H., Chen S.J., Ranasinghe D.C., Fumeaux C. (2021). A Frequency-Reconfigurable Wearable Textile Antenna With One-Octave Tuning Range. IEEE Trans. Antennas Propag..

[18] Dang Q.H., Chen S.J., Ranasinghe D.C., Fumeaux C. (2020). Modular Integration of a Passive RFID Sensor With Wearable Textile Antennas for Patient Monitoring. IEEE Trans. Compon. Packag. Manuf. Technol..

[19] Ashyap A.Y.I., Dahlan S.H., Abidin Z.Z., Dahri M.H., Majid H.A., Kamarudin M.R., Yee S.K., Jamaluddin M.H., Alomainy A., Abbasi Q.H. (2020). Robust and Efficient Integrated Antenna With EBG-DGS Enabled Wide Bandwidth for Wearable Medical Device Applications. IEEE Access.

[20] Atanasova G., Atanasov N. (2020). Small Antennas for Wearable Sensor Networks: Impact of the Electromagnetic Properties of the Textiles on Antenna Performance. Sensors.

[21] Atanasov N.T., Atanasova G.L., Atanasov B.N., Al-Rizzo H. (2020). Wearable Textile Antennas with High Body-Area Isolation:Design, Fabrication, and Characterization Aspects. Modern Printed Circuit Antennas.

[22] Simorangkir R.B.V.B., Yang Y., Matekovits L., Esselle K.P. (2017). Dual-Band Dual-Mode Textile Antenna on PDMS Substrate for Body-Centric Communications. IEEE Antennas Wirel. Propag. Lett..

[23] Simorangkir R.B.V.B., Kiourti A., Esselle K.P. (2018). UWB Wearable Antenna with a Full Ground Plane Based on PDMS-Embedded Conductive Fabric. IEEE Antennas Wirel. Propag. Lett..

[24] Al-Sehemi A., Al-Ghamdi A., Dishovsky N., Atanasova G., Atanasov N. (2020). Flexible polymer/fabric fractal monopole antenna for wideband applications. IET Microw. Antennas Propag..

[25] Memon A.W., de Paula I.L., Malengier B., Vasile S., Van Torre P., Van Langenhove L. (2021). Breathable Textile Rectangular Ring Microstrip Patch Antenna at 2.45 GHz for Wearable Applications. Sensors.

[26] Bait-Suwailam M.M., Labiano I.I., Alomainy A. (2020). Impedance Enhancement of Textile Grounded Loop Antenna Using High-Impedance Surface (HIS) for Healthcare Applications. Sensors.

[27] Elias N.A., Samsuri N.A., Rahim M.K.A., Othman N., Jalil M.E. Effects of human body and antenna orientation on dipole textile antenna performance and SAR. Proceedings of the 2012 IEEE Asia-Pacific Conference on Applied Electromagnetics (APACE).

[28] Rashid M.M.U., Rahman A., Paul L.C., Sarkar A.K. Performance Evaluation of a Wearable 2.45 GHz Planar Printed Meandering Monopole Textile Antenna on Flexible Substrates. Proceedings of the 2019 1st International Conference on Advances in Science, Engineering and Robotics Technology (ICASERT).

[29] Ashraf J., Jabbar A., Arif A., Riaz K., Zubair M., Mehmood M.Q. A Textile Based Wideband Wearable Antenna. Proceedings of the 2021 International Bhurban Conference on Applied Sciences and Technologies (IBCAST).

[30] Gao G., Yang C., Hu B., Zhang R., Wang S. (2019). A Wide-Bandwidth Wearable All-Textile PIFA With Dual Resonance Modes for 5 GHz WLAN Applications. IEEE Trans. Antennas Propag..

[31] Yang H., Azeez H.I., Wu C.-K., Chen W.-S. “Design of a fully textile dualband patch antenna using denim fabric”. Proceedings of the 2017 IEEE International Conference on Computational Electromagnetics (ICCEM).

[32] Bhardwaj P., Badhai R.K. Design and Analysis of Flexible Microstrip Antenna for Wearable Applications at ISM Band. Proceedings of the 2020 IEEE 17th India Council International Conference (INDICON).

[33] Sanz-Izquierdo B., Huang F., Batchelor J.C. (2006). Covert dual-band wearable button antenna. Electron. Lett..

[34] Sayem A.S.M., Esselle K.P. A Unique, Compact, Lightweight, Flexible and Unobtrusive Antenna for the Applications in Wireless Body Area Networks. Proceedings of the 2019 13th International Conference on Signal Processing and Communication Systems (ICSPCS).

[35] Mantash M., Tarot A.-C., Collardey S., Mahdjoubi K. Zip based monopole antenna for wearable communication systems. Proceedings of the 2012 6th European Conference on Antennas and Propagation (EUCAP).

[36] Alil S.M., Cheab S., Socheatra S., Jeoti V., Saeidi T., Abidin Z.Z. A Robust Wearable Unique-Logo Wideband Antenna for 5G Applications. Proceedings of the 2020 IEEE Student Conference on Research and Development (SCOReD).

[37] Li W., Chung K.L., Li Y. Characteristic Mode Design of Chanel-Logo Shaped Antenna. Proceedings of the 2020 IEEE 3rd International Conference on Electronic Information and Communication Technology (ICEICT).

[38] Monti G., Corchia L., Tarricone L. Logo antenna on textile materials. Proceedings of the 2014 44th European Microwave Conference.

[39] Shikder K., Arifin F. Extended UWB wearable logo textile antenna for body area network applications. Proceedings of the 2016 5th International Conference on Informatics, Electronics and Vision (ICIEV).

[40] Jørgensen K.L., Jakobsen K.B. Logo antenna for 5.8 GHz wireless communications. Proceedings of the 2016 International Workshop on Antenna Technology (iWAT).

[41] Tak J., Choi J. (2015). An All-Textile Louis Vuitton Logo Antenna. IEEE Antennas Wirel. Propag. Lett..

[42] Mahmud M.S., Dey S. Design, performance and implementation of UWB wearable logo textile antenna. Proceedings of the 2012 15 International Symposium on Antenna Technology and Applied Electromagnetics.

[43] Saha P., Mandal B., Chatterjee A., Parui S.K. Harmes Paris logo shaped wearable antenna for multiband applications. Proceedings of the 2016 Asia-Pacific Microwave Conference (APMC).

[44] Tumsare K.V., Zade P.L. Dual band logo antenna for WLAN application. Proceedings of the 2016 World Conference on Futuristic Trends in Research and Innovation for Social Welfare (Startup Conclave).

[45] Kiourti A., Volakis J.L. Wearable antennas using electronic textiles for RF communications and medical monitoring. Proceedings of the 2016 10th European Conference on Antennas and Propagation (EuCAP).

[46] Kiourti A., Volakis J.L. (2015). Colorful Textile Antennas Integrated into Embroidered Logos. J. Sens. Actuator Netw..

[47] Choi J.H., Kim Y., Lee K., Chung Y.C. Various wearable embroidery RFID tag antenna using electro-thread. Proceedings of the 2008 IEEE Antennas and Propagation Society International Symposium.

[48] Monti G., Corchia L., Tarricone L. Textile logo antennas. Proceedings of the 2014 Mediterranean Microwave Symposium (MMS2014).

[49] Monti G., Corchia L., De Benedetto E., Tarricone L. (2016). Wearable logo-antenna for GPS-GSM-based tracking systems. IET Microw. Antennas Propag..

[50] Balanis C.A. (2005). Antenna Theory: Analysis and Design.

[51] Liao C.-T., Yang Z.-K., Chen H.-M. (2021). Multiple Integrated Antennas for Wearable Fifth-Generation Communication and Internet of Things Applications. IEEE Access.

[52] Choi H.W., Shin D.W., Yang J., Lee S., Figueiredo C., Sinopoli S., Ullrich K., Jovančić P., Marrani A., Momentè R. (2022). Smart textile lighting/display system with multifunctional fibre devices for large scale smart home and IoT applications. Nat. Commun..

[53] Metaxas A.C., Meredith R.J. (2008). Industrial Microwave Heating.

[54] Rahmat-Samii Y., Guterman J., Moreira A.A., Peixeiro C., Balanis C. (2008). Integrated antennas for wireless personal communications. Modern Antenna Handbook.

[55] Fenn A.J. (2018). Electromagnetics and Antenna Technology.

[56] Sabban A. (2020). Wideband Wearable Antennas for 5G, IoT, and Medical Applications. Advanced Radio Frequency Antennas for Modern Communication and Medical Systems.

[57] Sharawi M.S., Hammi O. (2018). Design and Applications of Active Integrated Antennas.

[58] (2019). IEEE Standards for Safety Levels with Respect to Human Exposure to Electric, Magnetic, and Electromagnetic Fields, 0 Hz to 300 GHz.

[59] International Commission on Non-Ionizing Radiation Protection (ICNIRP) (2020). Guidelines for Limiting Exposure to Electromagnetic Fields (100 kHz to 300 GHz). Health Phys..

